# Sterilisation in Dentistry: A Review of the Literature

**DOI:** 10.1155/2019/6507286

**Published:** 2019-01-15

**Authors:** Enrica Laneve, Bruna Raddato, Mario Dioguardi, Giovanni Di Gioia, Giuseppe Troiano, Lorenzo Lo Muzio

**Affiliations:** Department of Clinical and Experimental Medicine, University of Foggia, Via Rovelli 50, Foggia 71122, Italy

## Abstract

In a small and medium-sized dental facility, the correct management of the sterilisation and presterilisation phases plays a fundamental role in good management of instruments and personnel, in order to ensure conditions that are more efficient with less down time. Nowadays, instrument sterilizers are increasingly efficient in achieving results, both in terms of time and size, and ensure that materials are sterile and ready to be stocked in a reasonable time. A literature search for articles related to revision work was performed using electronic databases such as PubMed, Scopus, and Google Scholar. The following keywords have been entered in the previously mentioned databases: sterilisation instruments; dental autoclave; precleaning; instruments disinfectants. The records obtained were screened by three reviewers, and only relevant articles were read full text. In addition, the timings of dental and sterilisation procedures were measured, and from these, suggestions are made in order to improve the efficiency of instrumentation management (facility used as study subject: University Dental Clinic, University of Foggia) as a function of the health-care interventions. We arrived at the conclusion that without doubt, sterilisation of instruments and products plays a fundamental role, but the efficiency of the sterilisation and presterilisation procedures cannot be separated from managing the personnel in charge by giving them specific and precise tasks.

## 1. Introduction

Increasingly, management of dental procedures requires more skilled dentists, in terms of both knowledge and competence [1, 2]. The speed of execution of a given intervention depends on factors that are related to the procedure underway, and the ability of the team to deal with critical situations, in managerial terms (late patients, overlaps in services, more consultants in the practice, and more investigative interventions) [3, 4]. In these situations, it is essential not to undermine both the correct daily dental practice methodology and the instruments sterilisation and disinfection procedures; hence, because of this, the capacity to be able to use sterile and well-stocked instruments in a reasonable time is priceless. To have a lot of sterile material in stock ready to use, it is essential to manage instruments properly and have the most efficient instruments and sterilizers [4–6].

Sterilisation is a procedure that destroys any living organism, pathogenic and nonpathogenic, in a vegetative form or spore present on the surface of the material to be sterilised [7]. An item or product that is free of living microorganisms is defined as sterile [8].

Sterilisation must be performed with a repeatable, standardisable, verifiable, and documentable method.

Chemical sterilisation is a method used for the decontamination of thermosensitive instruments, which cannot withstand cycles of autoclaving [9]. For the rest, autoclave sterilisation should be considered the elected procedure [10].

Over the years, the most common forms of physical heat sterilisations in dental practices have been saturated steam, chemical steam, and dry heat. The latter 2 methods are considered unreliable and of limited use [11].

The autoclave is the instrument responsible for the sterilisation of dental instruments [12]. At the start of the sterilisation cycle in the preliminary phase, a pump aspirates the air present in the sterilisation chamber. This phase is essential, because the air in the chamber acts as an insulating barrier which prevents uniform penetration and diffusion of the steam homogeneously within the instruments. A fractional vacuum phase distinguishes the latest generation autoclaves from the older generation [13].

After the air inside the chamber has been expelled, the steam is introduced: air-steam substitution takes place in several stages. At the end of the phase involving evacuation and substitution with steam, the pressure inside the chamber is higher than the atmospheric one, which leads to an increase in the boiling point of the water and, as a consequence, a hotter vapor [14, 15].

When the boiling temperature is reached, the materials inside the autoclave are left in contact with the steam for the predetermined time period needed to kill off all of the vegetative forms and living spores. After this period, the steam is expelled, and the material is vacuum-dried.

The last phase of the cycle involves restoring the sterilisation chamber pressure to the same level as the atmospheric one.

For the moment, the instrument to be put in storage is ready to be used for clinical/surgical use and can be used, until being reinserted and sterile, into a new clinical/surgical process [16, 17]. It undergoes a precise sequence of operations that must be carried out, which require a certain amount of time. Some factors are operator-related, such as time of execution of the procedure and time required by the assistants to properly organize the operative environment. Other factors are related to the time required by the autoclave to process an entire cycle of sterilisation.

The purpose of this review was to use literature and bibliographic sources to investigate the most up-to-date procedures, for ensuring optimisation of time and sterilisation. In addition, practical cases of managing instrument sterilisation and disinfection procedures and dental stations are described, focusing on controlling the execution time and human resources used.

## 2. Materials and Methods

The research method for the following review was performed in accordance with PRISMA guidelines [18]. All the studies that paid attention to sterilisation procedures and the management of dental instruments were taken into consideration.

The studies were identified by using electronic databases, examining the bibliography in the articles retrieved, and consulting experts in the field. No restrictions were applied to the language of publication, and articles in foreign languages were translated with the help of automatic translators (Google Translate). Bibliographic research was conducted on PubMed, Scopus, and Google Scholar. A complete overview of the search methodology, the keywords used, and the Boolean operator adopted is shown in Table 1.

The last search was conducted on April 25, 2018. The search and subsequent screening of the records obtained was conducted by two independent reviewers (EL; BR) while a third reviewer (MD) acted in the task of decision-making in doubtful situations. Screening included analysing the title and the abstract to eliminate records that were not relevant to the aims of the review; the overlaps were then eliminated. Following the guidelines of the PRISMA protocol, we defined the following inclusion and exclusion criteria to carry out the articles eligible for the qualitative analysis:

- Inclusion criteria: articles about those procedures preventing microbiological contamination; articles strictly related to the clinical and surgical dental activities; articles describing the contamination pathways in dental facilities; articles about new technologies integrated in dental facilities to reach a better decontamination rate
- Exclusion criteria: all those articles about decontamination and sterilisation issues that did not have clear connections with the dental environment.

Potentially eligible articles were finally submitted to a full-text analysis for verification via qualitative analysis; disputes were resolved by a fourth reviewer. Two auditors were in charge of research and screening: EL and GDG. The third auditor was GT; the fourth auditor, who had a supervisory task, was LLM. The practical cases for managing materials and dental staff were performed at the Dental Clinic of the University of Foggia, in collaboration with the staff responsible for assisting the dentist.

The facility has 14 dental stations and a sterilisation room located at the centre, where the times of the common daily dental procedures have been measured. All procedures were performed in a single dental station, by a single dentist, aided by two assistants, with a third one who measured their duration.

## 3. Results

**Figure 1:**
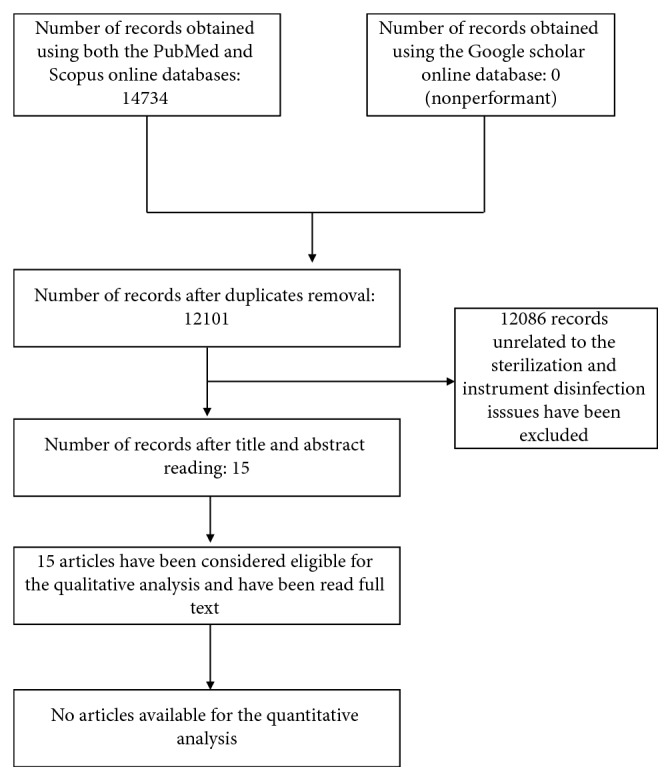
PRISMA flow diagram illustrating the process of selection of the eligible articles used in this paper.

The database search performed on PubMed, Scopus, and Google Scholar, the keywords used, and their combination using specific Boolean factors, including the number of articles obtained, are fully described in Figure 1, illustrating the PRISMA flow diagram. After the duplicates, removing all those articles unrelated to the issue of sterilisation and instruments disinfection, the total number of records was 400. After reading their titles and abstracts, 385 articles that did not have clear connections with the dental environment have been eliminated so that only 15 articles have been considered eligible for the qualitative analysis, and so they have been read full text (a complete overview of the 15 articles is shown in Table 2). Not a single article was available for the quantitative analysis. In addition, practical cases describing the time management of procedures for the study were performed.

Effective sterilisation and decontamination processes succeed in preventing cross infections and reduce microbiological degree of contamination in the operative environment [25].

The main infections that can be contracted in a dental setting are caused by bacteria such as *Staphylococcus aureus*, *Pseudomonas aeruginosa*, *Streptococcus pyogenes*, *Streptococcus pneumoniae*, *Clostridium tetani* (tetanus), *Legionella* [26, 27], mycobacteria such as *Mycobacterium tuberculosis* (TB) [28], fungi like *Candida albicans*, and viruses such as HIV (AIDS) [29, 30], HAV (type A hepatitis), HBV (hepatitis B), HCV (hepatitis C) [31], HDV (hepatitis D), HEV (hepatitis E), HGV (hepatitis G), HSV1/2 (herpes types 1 and 2), measles virus, mononucleosis (Epstein–Barr), varicella zoster, rubella, mumps, influenza, diphtheria, SARS, and prions, which cause spongiform encephalopathies [32, 33].

These infectious agents are present in the oral cavity and the respiratory system, and from there, they can enter the blood and saliva [33, 34]. The aerosol effect of some dental instruments favours the spread of these microorganisms, as phonation, sneezing, and coughing do [35, 36].

The transmission pathways for infectious diseases in a dental practice are of the “horizontal” type and can be from the patient to operator, from operator to patient, and from patient to patient [37–39].

Transmission occurs when pathogens come into direct contact with tissues, via a wound, blood, or secretions, by indirect contact with infected instruments, as well as by a consequence of aerosol formation, or water contamination by the creation of biofilm in water pipes [40–42].

To prevent patient-to-operator and operator-to-patient contamination, the dental team is equipped with barriers, which are defined as personal protective equipment (PPE): gloves [43], medical bonnets, masks, medical safety glasses, protective gowns, visors, screens, and aprons [44]. To avoid patient-to-patient transmission, the procedures implemented include using disinfectants and sterilising machines: autoclaves [45].

Disinfection and decontamination have to be put into practice not only to clean the instruments used in dental procedures [21–47] but also to clean surfaces that may have come into contact with the patient's biological fluids or been contaminated by aerosols, like the surfaces of the dental chair and dental carts [48]. Here, we list a series of the most commonly used disinfectants: formaldehyde; glutaraldehyde, peracetic acid, potassium peroxy-monosulphate complexes, phenols, alcohols, iodine compounds, chlorate compounds, quaternary ammonium salts, and chlorhexidine.

The disinfection procedures following the decontamination phase, meaning the physicochemical removal of infecting agents from instruments in order to lower the infection burden, are not enough to ensure all of the microorganisms responsible for cross infections are destroyed; in fact spores are resistant to the majority of disinfectants.

We necessarily therefore have to resort to sterilisation procedures [49].

## 4. Discussion

### 4.1. Practical Examples of Managing Instrumentation and Human Personnel

To provide practical examples of management, we refer to what happens every day on our own premises (at the dental clinic of the University of Foggia. This unit has as many as 14 dental stations available for clinical use). The times for executing the procedures were calculated by the authors and remeasured 3 times. The estimated times are the average of the 3 measurements.

**Figure 2:**
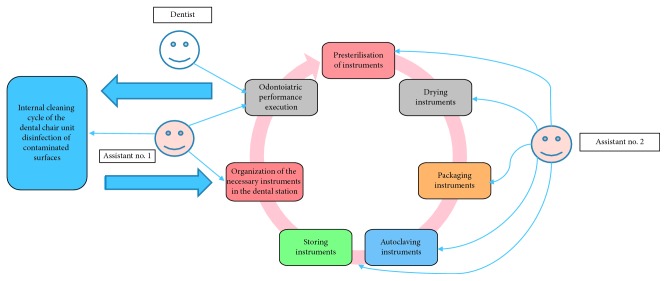
Sterilisation and disinfection cycle of dental instruments and dental station.

The case described involves the execution of Black's 1st Class filling, and the scenario recreated assumes there are 1 dental station, 2 assistants, and a dentist (Figure 2).

The instrumentation necessary for the execution are as follows: a carpule syringe, anaesthetic, a needle, 2 specimen tweezers, a dental dam sheet, a dental dam frame, clamp holder, rubber dam punch, tooth clamps, medium and fine grain diamond burs [23], tungsten carbide multiblade round burs rosettes, rubbers for polishing, adhesive systems, a photo-polymerising lamp, composite materials for reconstructions, microbrushes, air turbine high-speed handpiece, composite modelling instruments [50], a contrangle low-speed handpiece, air-water syringe, and articulation paper, including individual protection devices for the patient, operator, and assistant.

All these instruments go into different disinfection and sterilisation processes; the disposable ones are eliminated and disposed of; some are disinfected and cleaned (photo-polymerising lamp) [22], whilst others like handpieces and burs undergo a sterilisation process that is necessarily longer and have different timings [24].

  Step 1: the dental station was prepared, estimated time = 7 minutes (operation performed by both assistants).
  Step 2: time for the execution of the first-class filling = 30 minutes (with the aid of just 1 assistant). At the end of the second step, the dentist cannot influence anymore the timing of the entire sterilisation and disinfection session and so is ready for a new dental procedure.
  Step 3: the dental station box was decontaminated and disinfected (7 minutes); in addition, the instruments undergo a presterilisation phase of rinsing, drying, and packaging (20 minutes) and a sterilisation phase by autoclaving; the total estimated time is 1 hour and 30 minutes.

The above-described case leads to the conclusion that an instrument is ready to be used once more, since it is removed from its envelope discarded, in circa 2 hours, while the dentist performs Step 2 and is ready for a new filling after 30 minutes.

### 4.2. Suggestions to Improve the Working Efficiency in the Dental Facility

From this case, 4 findings can be deduced.

**Figure 3:**
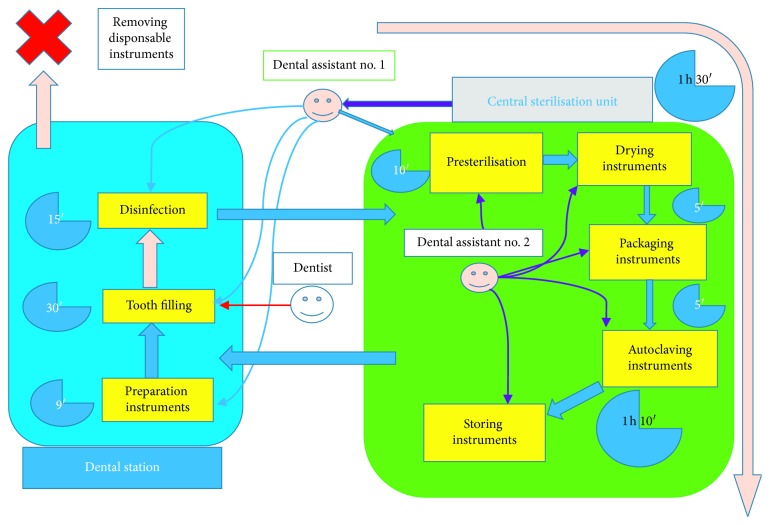
Sterilisation and disinfection cycle of dental instruments, with relative timings and tasks for each member of the dental team (Finding 1).

**Figure 4:**
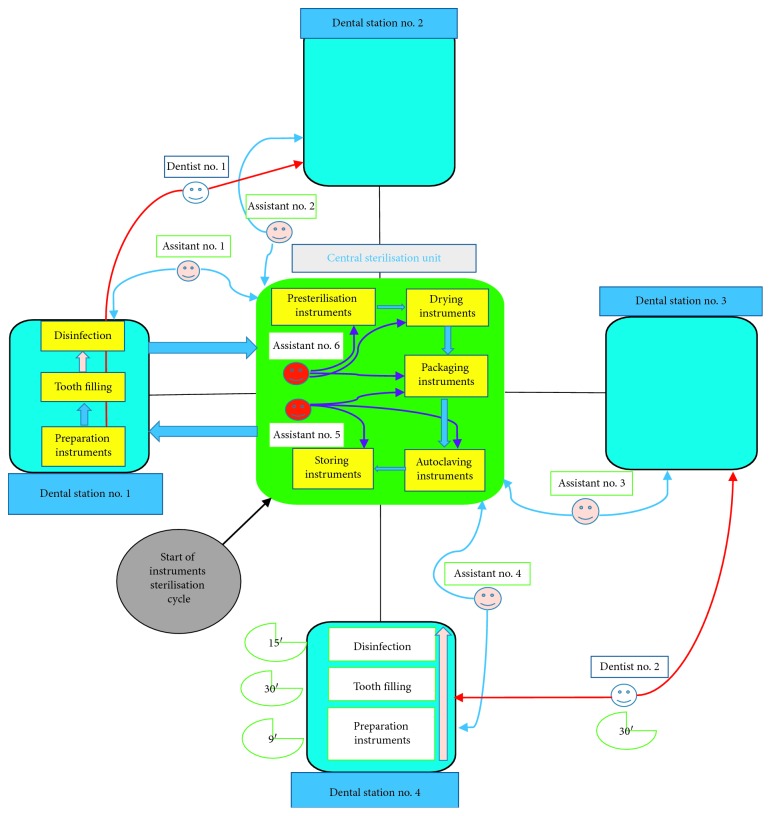
Sterilisation and disinfection cycle of dental instruments, with relative timings and tasks for each member of the dental team (Finding 3).

  Finding 1: Waiting for an instrument going into the sterilisation phase is definitely a loss of operative efficiency; in fact, in the same amount of time, the operator could perform other 3 procedures. Therefore, we can deduce that, after the execution of 4 fillings by 4 different dentists, considering the amount of time needed to completely sterilise the used instruments, we would have to wait no less than 2 hours. Every operator practicing in the conservative dentistry should have at least 4 complete sets of instruments at the beginning operative cycle.
  Finding 2: The use of 1 dental station by a single operator and the loss of efficiency of the individual dentist. The dental station undergoes an entire operative cycle: bringing the set of instruments to the dental station, executing the filling, cleaning the internal surfaces of the dental station, and waiting an internal cleaning cycle for the dental chair unit, in order to return to its initial state in 1 hour. Having 2 available dental stations will definitely reduce the amount of down time; in fact, while an assistant helps the dentist in performing dental procedures, the other one has all the time to bring the previously used instruments to the sterilisation room, cleanse, disinfect, and decontaminate the surfaces and then reorganize the dental station for the execution of a new filling in around 30 minutes, which gives the dentist the possibility to perform the next obturation without time loss (Figure 3).
  Finding 3: Having 2 assistants operating in a situation where one single dentist can work using 2 dental stations and 4 complete sets of instruments would lead to a bottleneck in the sterilisation procedure, since there would be a lack of personnel managing the sterilisation of nondisposable instruments. In this case, there would be the need of a third assistant located in the sterilisation room, whose task would be to start the autoclaving cycle, having a continuous availability of sterile instruments (Figure 4).
  Finding 4: On a theoretical level, in order to gain the maximum efficiency of the entire dental team, the dental facility would need no more than 7 dentists, working in 14 dental stations with at least 14 assistants, adding other personnel to the sterilisation room, whose number would need to be evaluated. At the beginning of every working day, every operator would be assigned at least 4 sets of instruments that will go through the entire sterilisation cycle.

## 5. Conclusions

Finally, in our opinion, a proper management of the sterilisation and disinfection procedures in an operating environment cannot be achieved without an efficient coordination within the dental team (dentists and assistants). According to our findings, only an appropriate workflow relationship between dentists, assistants, dental stations, and stored material can guarantee the maximum efficiency in terms of operative time, if the highest standard of instrument and operative environment sterility and disinfection are to be maintained.

## Figures and Tables

**Table 1:** A complete overview of the search methodology, illustrating the keywords used, the Boolean operators adopted, and the number of records obtained for each online database.

	PubMed	Scopus	Google Scholar
“Sterilisation instruments” AND “dental autoclave”	134	0	117
“Sterilisation instrument” AND “precleaning”	0	0	1
“Sterilisation instruments” AND “instruments disinfectants”	1270	0	1
“Dental autoclave” AND “precleaning”	1	0	0
“Dental autoclave” AND “instruments disinfectants”	32	0	0
“Precleaning” AND “instruments disinfectants”	2	1	1
“Sterilisation instruments”	7979	14	522
“Precleaning”	62	382	30500
“Instruments disinfectants”	4530	28	35600
“Dental autoclave”	297	2	16300
Total number of records	14307	427	83042

**Table 2:** The complete list of the 15 articles eligible for the qualitative analysis, with an appropriate description of their topics and their results.

Author(s) and date of publication	Title	Paper	Type of study	Topic/results of the study
Condrin, 2014 [1]	Disinfection and Sterilisation in Dentistry	Texas Dental Journal	Review	Review of the literature

Arancegui, 1994 [3]	Biological Safety in Dentistry: Development of a Useful Method for Quality Control of Sterilisation	Revista Argentina de Microbiología	In vitro study	534 autoclave cycles tested. The results showed that 86.90% of the autoclaves lacked thermometers, 76.60% lacked manual thermostats, 83.33% were automatic and 58.80% did not sterilise

Ling, 2018 [5]	APSIC Guidelines for Disinfection and Sterilisation of Instruments in Health-Care Facilities	Antimicrobial Resistance & Infection Control	Guidelines	Guidelines for disinfection and sterilisation of instruments in health-care facilities

Chidambaranathan, 2017 [6]	Comprehensive Review and Comparison of the Disinfection Techniques Currently Available in the Literature	Journal of Prosthodontics	Review	This article critically analyzes the various published methods of dental impression disinfection in dentistry

Lacerda, 2015 [9]	Evaluation of Two Disinfection/Sterilisation Methods on Silicon Rubber-Based Composite Finishing Instruments	American Journal of Dentistry	In vitro study	Both sterilisation/disinfection methods were efficient against oral cultivable organisms, and no deleterious modification was observed to point surface

Costa, 2017 [11]	Alcohol Fixation of Bacteria to Surgical Instruments Increases Cleaning Difficulty and May Contribute to Sterilisation Inefficacy	American Journal of Infection Control	In vitro study	Treating contaminated instruments with alcohol, drying them, or soaking them in water for prolonged periods increases cleaning difficulty and should be discouraged

Healy, 2004 [19]	Autoclave Use in Dental Practice in the Republic of Ireland	International Dental Journal	In vitro study	To assess, by postal questionnaire, cross-infection control methods, especially sterilisation procedures, of 700 general dental practitioners in the Republic of Ireland and to biologically monitor steam pressure sterilizers or autoclaves in their practices

Edwardsson, 1983 [20]	Steam Sterilisation of Air-Turbine Dental Handpieces	Acta Odontologica Scandinavica	In vitro study	The results indicate that the instrument autoclaves with built-in programs for 120–124°C/20 min and 134–136 C/10 min could have insufficient capacity to sterilise lubricated or unlubricated dental handpieces

Andersen, 1999 [15]	Effect of Steam Sterilisation inside the Turbine Chambers of Dental Turbines	Oral Surgery, Oral Medicine, Oral Pathology, and Oral Radiology	In vitro study	Results indicate that cleaning before sterilisation is essential for safe use of high-speed dental turbines and that small nonvacuum autoclaves should be carefully evaluated before being used for the reprocessing of hollow instruments such as high-speed turbines

Palenik, 1994 [16]	Effectiveness of Two Types of Sterilisation on the Contents of Sharps Containers	American Journal of Dentistry	In vitro study	The purpose of this study was to evaluate the killing effect that a gravity steam autoclave or a high-vacuum steam sterilizer or an unsaturated chemical vapor sterilizer had on endospores present on strips or their effect if applied to dental needles within three sizes of sharps containers

Sheldrake, 1995 [17]	Effectiveness of Three Types of Sterilisation on the Contents of Sharps Containers	Quintessence International	In vitro study	The purpose of this study was to test the effect of treatment in a gravity steam autoclave, high-vacuum steam autoclave, or an unsaturated chemical vapor sterilizer on endospores present on strips or placed inside of dental anaesthetic cartridges held within sharps containers

Thomas, 2005 [46]	Methods of Dental Instrument Processing, Sterilisation, and Storage: A Review	Texas Dental Journal	Review	Review of the literature
Smith, 2007 [22]	Sterilisation of ReUsable Instruments in General Dental Practice	British Dental Journal	Review	To examine the methods used for sterilisation of reusable instruments in general dental practice, including the installation, commissioning, and testing of benchtop steam sterilizers

Mathivanan, 2017 [23]	Evaluation of Efficiency of Different Decontamination Methods of Dental Burs: An In Vivo Study	Journal of Pharmacy and Bioallied Sciences	In vitro study	The present study was done to quantitatively and qualitatively assess the pathogenic contamination of dental burs used for tooth preparation and to determine the effective method of sterilisation (autoclave, glass bead sterilizer, hot air oven, and surgical spirit immersion) of burs used for crown preparation

Apinhasmit, 2003 [24]	Effects of Autoclave Sterilisation on Properties of Dental Rubber Dam as Related to Its Use as Barrier Membrane in Guided Tissue Regeneration	Journal of Periodontal Research	In vitro study	These results suggest that the autoclave sterilisation deteriorated the physical properties of rubber dams even though they seemed to be compatible to the cultured human cells. Therefore, the sterilisation method should be taken into consideration when rubber dams are utilized as barrier membranes
